# Nanoperlite Particles Enhance Fibrogenesis in Thyroid Orbital Fibroblasts: A Potential Activated Cell Source for Ocular Tissue Engineering

**DOI:** 10.1155/bmri/1795961

**Published:** 2026-03-08

**Authors:** Fatemeh Sanie-Jahromi, Razi Sahraeian, Behzad Khademi

**Affiliations:** ^1^ Poostchi Ophthalmology Research Center, Department of Ophthalmology, School of Medicine, Shiraz University of Medical Sciences, Shiraz, Iran, sums.ac.ir; ^2^ Composites Department, Faculty of Processing, Iran Polymer and Petrochemical Institute, Tehran, Iran, ippi.ac.ir

**Keywords:** fibrogenesis, nanoperlite, orbital fibroblasts (OFs), periorbital tissue engineering, thyroid eye disease (TED)

## Abstract

Fibroblasts are crucial in tissue engineering because of their ability to synthesize the extracellular matrix (ECM) and secrete growth factors. Orbital fibroblasts (OFs) from patients with thyroid eye disease (TED) exhibit enhanced fibroblastic properties, making them ideal candidates for regenerative medicine in ocular tissue. In the present study, we investigated the effect of nanoperlite on TED OFs. Nanoperlite, with its unique properties including high silica (SiO_2_) content, holds promise for enhancing fibroblast functions. Nanoperlite was prepared and characterized in terms of particle size and chemical composition. A sample of orbital adipose tissue was taken from a TED patient during orbital decompression surgery and OFs were expanded in vitro. The cells were then treated with nanoperlite at concentrations of 1 and 10 *μ*g/mL for 24 h, and gene expression related to the fibrogenesis process was assessed using real‐time PCR. Nanoperlite at 1 *μ*g/mL significantly increased the expression of TGF‐*β*, CD90, *α*‐SMA, ZEB1, *β*‐Catenin, and Snail genes in OFs. However, at 10 *μ*g/mL, this effect was not observed. This study highlights nanoperlite′s potential to enhance fibroblast activity specifically at the concentration of 1 *μ*g/mL. This effect can potentially aid tissue engineering strategy for periorbital tissue repair and eyelid reconstruction. However, further research is needed to fully elucidate its therapeutic potential and safety profile.

**Key Messages**

**What Is Known:**

• Fibroblasts play a crucial role in tissue engineering due to their ability to synthesize the extracellular matrix (ECM) and secrete growth factors, with orbital fibroblasts (OFs) from thyroid eye disease (TED) patients showing enhanced fibroblastic traits.

**New Information:**

• Nanoperlite, with its high silica (SiO_2_) content, has the potential to enhance fibroblast functions, particularly in OFs from TED patients.
• At a concentration of 1 *μ*g/mL, nanoperlite significantly increases the expression of key fibrogenesis‐related genes (TGF‐*β*, CD90, *α*‐SMA, ZEB1, *β*‐Catenin, and Snail) in TED OFs, potentially aiding tissue engineering strategies for periorbital tissue repair and eyelid reconstruction.
• The effect of nanoperlite on gene expression is concentration‐dependent, with 10 *μ*g/mL not producing the same enhancement, indicating the importance of dosage in its application.

## 1. Introduction

Fibroblasts are a type of mesenchymal cells known for their ease of culture in laboratory settings [1]. Fibroblasts are present in several types of tissues including skin, adipose, and connective tissues [2]. These cells play a pivotal role in tissue engineering and wound healing [3, 4]. Not only do they secrete a variety of growth factors and cytokines, but they are also instrumental in producing the ECM and cell substrates. Their ability to generate an appropriate ECM and releasing growth factors makes them an ideal platform for supporting and proliferating epithelial cells [5, 6].

These cells are found in various tissues and, despite their similar morphology, exhibit unique gene expression profiles based on their anatomical location [7, 8]. They synthesize ECM proteins and cytokines in a location‐specific manner [8]. The activation of fibroblasts is essential for the secretion of factors and ECM proteins that facilitate wound healing [9–11]. This is particularly significant in the context of developing cell substrates for transplantation and skin tissue engineering, specifically for repairing extensive and chronic wounds such as eyelid injuries and oculoplastic reconstruction.

In the current research, we utilized OFs from patients with TED. These cells are typically discarded during orbital decompression surgery. TED is characterized by significant orbital tissue remodeling and fibrosis, leading to an inherently activated fibroblast phenotype in OFs from these patients [12, 13]. This activated state, characterized by heightened ECM production and profibrotic signaling, makes them a suitable model for studying agents that further enhance fibrogenesis for reconstructive purposes, as their baseline characteristics already align with the desired phenotype for tissue repair applications. This study investigated the potential of nanoperlite to enhance the fibroblastic characteristics of these cells. Perlite, an amorphous volcanic glass found naturally, is widely used in the polymer industry as a filler. Nanoperlite offers unique properties such as low cost, lightweight, high‐water content, fire resistance, and high silica (SiO_2_) content rendering it significant biological properties [14, 15]. Recent studies have shown the ability of SiO_2_ to enhance fibroblast proliferation and promote ECM deposition [16, 17]. These augmented fibroblasts hold promise as a valuable resource for creating a fibrotic substrate, crucial for supporting the skin epithelial layer in regenerative medicine approaches.

In this study, key markers of fibrogenesis pathway activation were investigated, including TGF‐*β*, CD90, *α*‐SMA, ZEB1, *β*‐Catenin, and Snail. TGF‐*β* plays a pivotal role by inducing fibroblast differentiation into myofibroblasts and increasing ECM synthesis [18]. CD90 is effective in cell interactions and fibroblast migration [19], and *α*‐SMA is a marker of myofibroblast differentiation and increased cell contractility [20]. ZEB1 and Snail, as transcription factors, enhance the expression of mesenchymal genes and cell migration [21–23]. *β*‐Catenin, a key component of the WNT signaling pathway, regulates cell proliferation, fibroblast activation, and ECM production [24].

This study offers new avenues for the treatment of extensive and chronic wounds, including those resulting from eyelid injuries.

## 2. Methods

### 2.1. Nanoperlite Preparation and Characterization

We purchased perlite particles, with an average size of approximately 7 *μ*m, from Varesh Sabz Dorfak, Iran. To achieve nanoscale dimensions (below 1000 nm), we employed a two‐step milling process. Initially, micronized perlite, ranging from 200 to 7000 nm, underwent three passes in a pinmill (CONDUX, D2800BREMEN). Subsequently, a ballmill (RETSCH, PM100) further reduced the particle size over 60 h at 60 rotations per minute. Particle size analysis was conducted using a laser light scattering device (SEMATECH, SEM‐663), following ASTM 301070 standards. Additionally, the Brunauer–Emmett–Teller (BET) was performed to evaluate the surface area of nanoparticles by nitrogen adsorption test (Micromeritics ASAP 2010 analyzer, Micromeritics Instrument). TEM micrographs and chemical composition analyses of the nanoperlite were also performed.

### 2.2. TED OFs Cell Culture

Orbital adipose tissue was extracted from a 37‐year‐old male patient with TED during orbital decompression surgery and stored under sterile conditions at 4°C until transferred to the cell laboratory. All methods were carried out in accordance with relevant guidelines and regulations and informed consent was obtained from the patient. TED OFs were cultured using the explant culture method. Briefly, the specimen was rinsed in phosphate‐buffered saline (PBS, Pars Tous, Iran), segmented into small pieces, and cultured in a complete growth medium. This medium was maintained in 5% CO_2_ at 37°C with saturated relative humidity. The growth medium consisted of Dulbecco′s Modified Eagle Medium (DMEM)/F12 (Shellmax, Iran) supplemented with 10% fetal bovine serum (FBS, Gibco, Germany) and 1% penicillin–streptomycin solution (P/S, Shellmax, Iran). Cell trypsinization and passage occurred at 80%–90% confluence, with media changes every 3 days.

### 2.3. Treatment With Nanoperlite

Nanoperlite was administered at concentrations of 1 and 10 *μ*g/mL to treat OFs. The cells from passages 5–7 were prepared and cultured at a density of 10^6^ cells per 10 cm^2^ of culture surface and treated with nanoperlite for 24 h. Control plates were incubated with the vehicle medium (without nanoperlite) under identical conditions.

**Figure 1:**
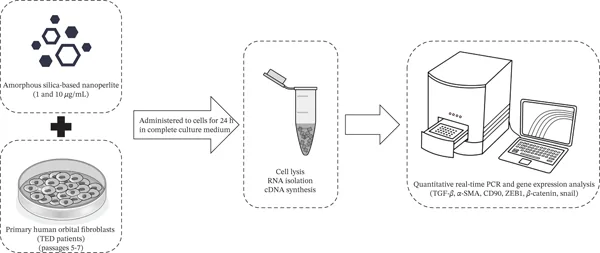
Workflow of thyroid orbital fibroblasts (TED OFs) treatment with nanoperlite. The TED OFs from passages 5–7 were cultured and treated with nanoperlite at concentrations of 1 and 10 *μ*g/mL for 24 h. Control plates received vehicle medium. Total RNA was extracted, and cDNA synthesis was performed for gene expression analysis.

After treatment, cell density and nanoparticle intake were assessed across the culture surface using phase‐contrast microscopy. Total RNA was then extracted using the RNeasy kit (Pars Tous, Iran), and cDNA synthesis was performed with the Easy cDNA Synthesis Kit (Pars Tous, Iran), according to the manufacturer′s instructions (Figure 1).

### 2.4. Gene Expression Analysis

Real‐time PCR was used for gene expression analysis. Primers for the target genes TGF‐*β*, CD90, *α*‐SMA, ZEB1, *β*‐Catenin, Snail, and *β*‐ACTIN were designed using AlleleID software Version 7.5. *β*‐ACT served as the housekeeping gene (Table 1). The PCR cycling conditions included initial hold (95°C for 15 min), denaturation (95°C for 10 s), annealing and extension (61°C for 40 s). The relative expression levels of the genes were quantified using the 2^−*ΔΔ*Ct^ method.

**Table 1:** The primer sequences of the genes under study.

Gene	Sense primer	Antisense primer	Product size
TGF‐*β*	GCAACAATTCCTGGCGATACC	CCTCAATTTCCCCTCCACGG	123 bp
CD90	CTTCACTAGCAAGGACGAGGG	ACCAGTTTGTCTCTGAGCACT	105 bp
ZEB1	CGCAGTCTGGGTGTAATCGT	TTCTTGGTCGCCCATTCACA	216 bp
*β*‐Catenin	CCGAATGTCTGAGGACAAGCC	TCAAGTCCAAGATCAGCAGTCTCA	117 bp
Snail	TAGCGAGTGGTTCTTCTGCG	CTGCTGGAAGGTAAACTCTGGAT	160 bp
*α*‐SMA	CACGATGTACCCTGGGATCG	GCGGGGCGATGATCTTGA	89 bp
*β*‐ACT	GCCTCGCCTTTGCCGAT	CATGCCGGAGCCGTTGT	98 bp

### 2.5. Bioinformatics Analysis of Gene Network

To further investigate the potential mechanistic pathways underlying the effects of nanoperlite, the studied genes (TGF‐*β*, CD90, *α*‐SMA, ZEB1, *β*‐Catenin, and Snail) and proteins associated with the TGF‐*β*/SMAD, WNT/*β*‐Catenin, and MAPK/PI3K/AKT pathways were subjected to protein–protein interaction (PPI) network analysis using the STRING database (http://string-db.org, Version 12.0). The analysis was performed for Homo sapiens with a confidence score threshold of 0.4. Interaction sources included text mining, experimental data, curated databases, coexpression, neighborhood, gene fusion, and co‐occurrence. The network was analyzed in Cytoscape software (Version 3.10.4) with topology parameters, including average shortest path length, betweenness centrality, and node degree, were calculated to assess the structural properties of the resulting network. MCODE analysis was also performed to identify the active clusters of the network and potential mechanistic links of the signaling cascades.

### 2.6. Statistical Analysis

Data were presented as mean ± standard error of mean (SEM) and analyzed using IBM SPSS Statistics 22 software. The one‐way ANOVA, followed by LSD post hoc tests, were applied to compare groups and determine the significance of differences observed. A *p* value of less than 0.05 was considered statistically significant.

## 3. Results

### 3.1. Nanoperlite Size and Chemical Composition

The dynamic light scattering (DLS) analysis confirmed an average size below 1000 nm (mean diameter from 200–500 nm) for nanoperlite particles (Figure 2a,b). BET analysis showed a mean surface area of 8.22 m^2^/g for nanoperlite particles (Figure 2c). The TEM electron microscopy of nanoperlite is represented in Figure 2d. The chemical composition analysis of the nanoperlite revealed that over 70% of its composition consists of SiO_2_. The remaining mass primarily comprises alumina (Al2O3), potassium oxide (K2O), and water.

Figure 2Characterization of nanoperlite particles. (a, b) Dynamic light scattering (DLS) analysis showing the size distribution of nanoperlite particles. (c) Brunauer–Emmett–Teller (BET) analysis of mean surface area of nanoperlite particles. (d) Transmission electron microscopy (TEM) image of nanoperlite particles, illustrating their spherical morphology and nanoscale dimensions in the absence of cells. This panel is intended to show the physical characteristics of the particles themselves, not their interaction with cells.

**Figure:**
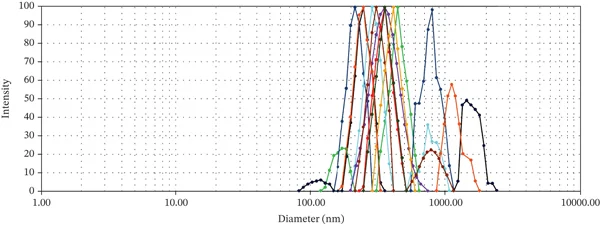
(a)

**Figure:**
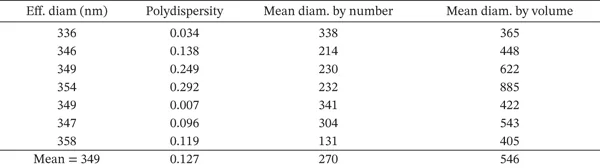
(b)

**Figure:**
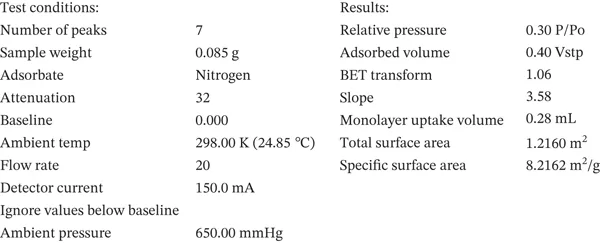
(c)

**Figure:**
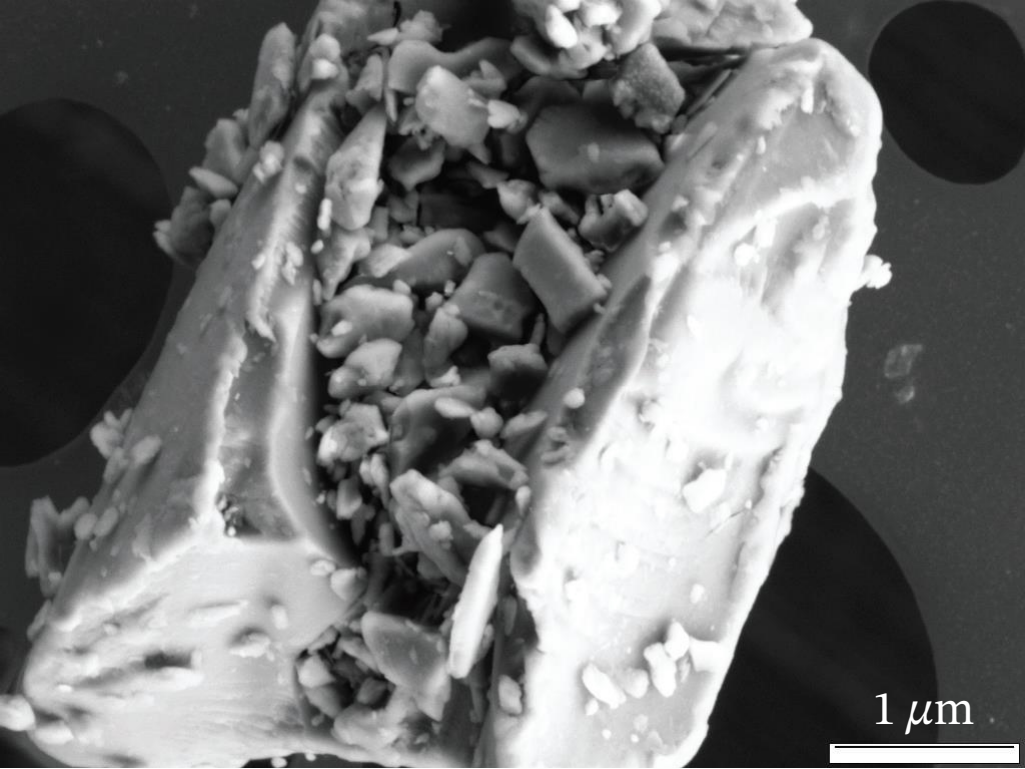
(d)

### 3.2. TED OF Characterization and Intake of Nanoperlite

**Figure 3:**
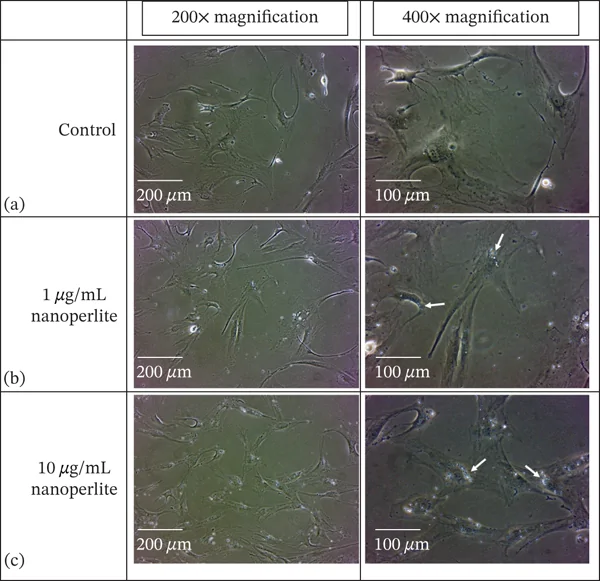
Morphological assessment and nanoperlite uptake in thyroid orbital fibroblasts (TED OFs). (a) Representative image of TED OFs showing typical flattened, elongated morphology with multiple cytoplasmic extensions under inverted phase contrast microscopy. (b, c) Inverted phase contrast microscopy images illustrating the distribution and uptake of nanoperlite particles in TED OFs after 24‐h incubation. Nanoparticle accumulation is more prominent around the perinuclear region in cells treated with 10 *μ*g/mL compared with 1 *μ*g/mL. White arrows highlight areas of increased particle density adjacent to the nuclei, suggesting internalization or surface association of nanoperlite.

TED OFs exhibited typical fibroblastic morphology (Figure 3a). We examined the distribution and uptake of nanoperlite particles in cells after a 24‐h incubation period. These nanoparticles, capable of reflecting light, allowed for effective tracking under a phase contrast microscope. The recorded images demonstrated successful uptake of nanoparticles by the cells, with the particles predominantly distributed around the cell nuclei. The density of absorbed nanoparticles was notably higher in cells treated with 10 *μ*g/mL of nanoperlite compared with those treated with 1 *μ*g/mL (Figure 3b,c).

### 3.3. Nanoperlite Activates TED OFs

**Figure 4:**
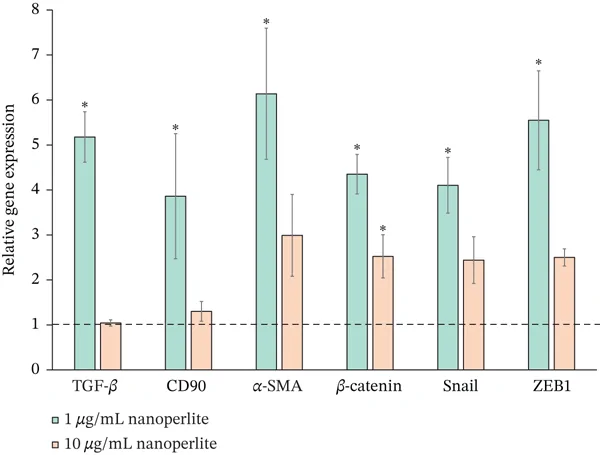
Nanoperlite induces fibrogenesis‐related gene expression in thyroid orbital fibroblasts (TED OFs). Relative gene expression of genes involved in fibrogenesis in TED OFs treated with 1 *μ*g/mL and 10 *μ*g/mL nanoperlite for 24 h compared with controls (dotted line, set as 1). Experiments were conducted in triplicate, and data are presented as mean ± SEM. Statistical significance was determined using one‐way ANOVA followed by LSD post hoc tests, with significance denoted as  ^∗^(*p* < 0.05). The findings reveal a dose‐dependent response, with distinct patterns of gene upregulation observed at different nanoperlite concentrations.

Our results demonstrated that nanoperlite induced a dose‐dependent modulation of fibrogenesis‐associated gene expression in TED OF cells. Treatment with 1 *μ*g/mL nanoperlite led to a marked upregulation of all fibrogenesis related genes compared with control cells (set as 1). Specifically, the expression levels of TGF‐*β* (5.18 ± 0.56, *p* < 0.001; *F*[2, 6] = 54.570), CD90 (3.86 ± 1.39, *p* = 0.047; *F*[2, 6] = 3.754), *α*‐SMA (6.14 ± 1.46, *p* = 0.011; *F*[2, 6] = 6.820), *β*‐Catenin (4.35 ± 0.44, *p* < 0.001; *F*[2, 6] = 19.822), Snail (4.10 ± 0.62, *p* = 0.003; *F*[2, 6] = 11.057), and ZEB1 (5.55 ± 1.10, *p* = 0.002; *F*[2, 6] = 13.016) were significantly increased (Figure 4).

In contrast, exposure to 10 *μ*g/mL nanoperlite resulted in a more limited response, with significant overexpression detected only for *β*‐Catenin (2.52 ± 0.48, *p* = 0.029; *F*[2, 6]: 19.822) (Table S1).

### 3.4. Bioinformatic Network Analysis Reveals Key Hubs in Fibrogenic Pathways

**Figure 5:**
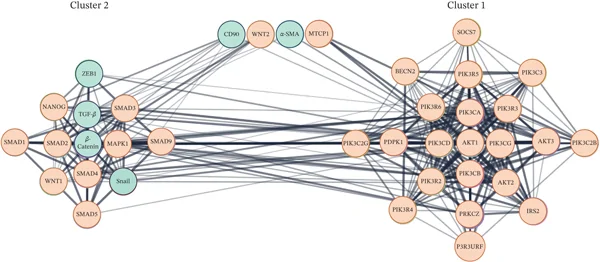
Protein–protein interaction (PPI) network of upregulated genes and other associated proteins involved in the TGF‐*β*/SMAD, WNT/*β*‐Catenin, and MAPK/PI3K/AKT signaling pathways. Two major functional clusters were identified: Cluster 1 (21 nodes, 176 edges; score = 17.6) and Cluster 2 (13 nodes, 63 edges; score = 10.5). The studied genes are shown as green nodes, whereas the associated proteins are shown as orange nodes. Notably, *β*‐Catenin, TGF‐*β*, Snail, and ZEB1 grouped within Cluster 2, forming a tightly connected regulatory hub.

The PPI network constructed from the upregulated genes and their associated proteins involved in the TGF‐*β*/SMAD, WNT/*β*‐Catenin, and MAPK/PI3K/AKT signaling pathways revealed two major functional clusters (Cluster 1 and Cluster 2) comprising highly interconnected nodes (Figure 5). Cluster 1 included 21 nodes and 176 edges (score = 17.6), whereas Cluster 2 comprised 13 nodes and 63 edges (score = 10.5).

From the studied genes, *β*‐Catenin, TGF‐*β*, Snail, and ZEB1 were grouped in Cluster 2, forming a tightly connected regulatory hub. Among these, *β*‐Catenin exhibited the highest degree (21) and betweenness centrality (0.044), identifying it as the central node of this cluster. TGF‐*β*, Snail, and ZEB1 followed with degrees of 19, 13, and 10, respectively, highlighting their key modulatory roles within this signaling module. The coexistence of WNT, SMAD, and MAPK pathway proteins within this cluster suggests that the upregulation of these genes may enhance the cross talk between the TGF‐*β*/SMAD, WNT/*β*‐Catenin, and MAPK signaling cascades.

Cluster 1 was primarily enriched in PI3K/AKT pathway components, centered around AKT1 (degree = 34, betweenness centrality = 0.254), which emerged as a major hub connecting multiple PI3K subunits (PIK3CA, PIK3CB, PIK3CD, PIK3CG, PIK3R2, PIK3R3, etc.). The close interconnection observed between Cluster 1 and Cluster 2 indicates that activation of the studied genes may simultaneously trigger the TGF‐*β*/SMAD and WNT/*β*‐Catenin pathways and further link them to PI3K/AKT signaling components, thereby amplifying the overall cellular response to nanoperlite exposure.

Although CD90 and *α*‐SMA were not assigned to either cluster, their relatively high neighborhood connectivity values (19.17 and 23.67, respectively) suggest that they may function as peripheral yet influential nodes, potentially bridging ECM remodeling processes with intracellular signaling pathways.

Collectively, these findings indicate that nanoperlite exposure activates a complex, multipathway signaling network dominated by the *β*‐Catenin/TGF‐*β* axis and its downstream convergence with PI3K/AKT signaling, which together may underlie the observed fibrogenic cellular responses (see Table 2 for detailed network topological parameters).

**Table 2:** Topological parameters of the protein–protein interaction network.

Name	BC	CC	CCo	Degree	Eccentricity	MCODE	NC	Radiality	Stress	TCo
AKT1	0.254	0.925	0.405	34	2	Cluster 1	15.353	0.998	1212	
PIK3CA	0.075	0.787	0.570	27	2	Cluster 1	17.852	0.992	548	
PIK3CG	0.017	0.712	0.740	22	2	Cluster 1	18.818	0.988	228	
PIK3R3	0.012	0.698	0.771	21	2	Cluster 1	18.905	0.987	178	0.511
PIK3CD	0.018	0.698	0.757	21	2	Cluster 1	18.810	0.987	188	
PIK3CB	0.011	0.698	0.781	21	2	Cluster 1	18.762	0.987	158	
*β*‐Catenin	0.044	0.698	0.519	21	2	Cluster 2	15.476	0.987	366	0.418
AKT2	0.027	0.685	0.626	20	2	Cluster 1	19.000	0.986	214	
PIK3R2	0.007	0.673	0.821	20	3	Cluster 1	18.850	0.986	108	0.524
PIK3R6	0.007	0.673	0.821	20	3	Cluster 1	18.850	0.986	108	
PIK3R5	0.007	0.673	0.821	20	3	Cluster 1	18.850	0.986	108	
TGF‐*β*	0.036	0.673	0.509	19	2	Cluster 2	13.895	0.986	244	0.376
PIK3C3	0.010	0.661	0.817	18	2	Cluster 1	19.111	0.985	126	
MAPK1	0.030	0.661	0.601	18	2	Cluster 2	17.611	0.985	252	
SMAD4	0.026	0.661	0.575	18	2	Cluster 2	15.611	0.985	238	0.422
SMAD2	0.024	0.661	0.575	18	2	Cluster 2	14.167	0.985	200	0.383
AKT3	0.013	0.649	0.735	17	2	Cluster 1	19.588	0.984	128	
PRKCZ	0.006	0.638	0.817	16	2	Cluster 1	20.750	0.983	62	0.561
IRS2	0.003	0.638	0.883	16	2	Cluster 1	20.375	0.983	36	0.551
PIK3R4	0.002	0.627	0.942	16	3	Cluster 1	19.563	0.983	46	0.543
PDPK1	0.002	0.627	0.914	15	2	Cluster 1	20.733	0.983	24	
BECN2	0.001	0.617	0.981	15	3	Cluster 1	19.867	0.982	36	0.552
SOCS7	0.001	0.617	0.981	15	3	Cluster 1	19.867	0.982	36	0.552
PIK3C2B	0.001	0.617	0.981	15	3	Cluster 1	19.867	0.982	36	
P3R3URF	0.000	0.536	1.000	14	3	Cluster 1	18.929	0.975	0	0.676
PIK3C2G	0.000	0.536	1.000	14	3	Cluster 1	18.929	0.975	0	
SMAD3	0.007	0.617	0.758	14	2	Cluster 2	16.000	0.982	94	0.432
Snail	0.004	0.607	0.782	13	2	Cluster 2	17.308	0.981	46	0.468
ZEB1	0.001	0.578	0.933	10	2	Cluster 2	18.400	0.979	14	0.497
WNT1	0.001	0.561	0.911	10	3	Cluster 2	16.300	0.977	8	0.466
NANOG	0.001	0.561	0.911	10	3	Cluster 2	16.300	0.977	8	0.466
SMAD9	0.001	0.552	0.972	9	3	Cluster 2	17.667	0.976	18	0.505
SMAD5	0.001	0.552	0.972	9	3	Cluster 2	17.667	0.976	18	0.505
SMAD1	0.000	0.487	1.000	8	3	Cluster 2	15.750	0.969	0	0.583
WNT2	0.000	0.529	1.000	6	3		20.000	0.974	0	
CD90	0.000	0.529	1.000	6	3		19.167	0.974	0	0.548
MTCP1	0.000	0.500	1.000	3	3		23.667	0.971	0	0.696
*α*‐SMA	0.000	0.507	1.000	3	3		23.667	0.971	0	

Abbreviations: BC, betweenness centrality; CC: closeness centrality; CCo: clustering coefficient; NC: neighborhood connectivity; TCo: topological coefficient.

## 4. Discussion

Activated fibroblasts are recognized for their increased ability to produce ECM and secrete growth factors, making them valuable for tissue engineering applications and skin regeneration in damaged tissues [25, 26]. Despite advances in the development of scaffolds for repairing damaged skin [27], skin regeneration around the eyes and eyelids remains a unique challenge [28]. This study employed OFs obtained from a patient with TED, which demonstrate an augmented fibrotic phenotype—a consequence of TED pathology [29].

Decompression surgery serves as a key strategy in the management of TED, aimed at addressing vision‐threatening optic nerve damage, corneal exposure, and cosmesis [30]. During this procedure, surgeons remove the fibrotic tissue which includes OFs. This tissue is commonly discarded, although it holds promise for tissue engineering applications. Given its orbital origin and the properties of the periorbital region, this tissue may offer an optimal combination of ECM and growth factors to support epithelial regeneration, particularly beneficial for ocular skin regeneration or eyelid reconstruction.

Our research aimed to enhance the fibrotic features of TED OFs and the expression of fibroblastic and myofibroblastic markers through the introduction of perlite nanoparticles, focusing on manipulating the behavior of TED OFs using nanotechnology.

Our findings demonstrated that nanoperlite significantly upregulated the expression of fibrogenesis‐related genes in OFs, with the most pronounced effect observed at 1 *μ*g/mL. Key markers, including TGF‐*β*, CD90, *α*‐SMA, ZEB1, *β*‐Catenin, and Snail, showed substantial elevation. TGF‐*β*, a central profibrotic cytokine, drives fibroblast‐to‐myofibroblast differentiation and enhances ECM components deposition like collagen [31]. TGF‐*β* also stimulates the production of other fibrosis‐associated proteins and inhibits ECM degradation, thus contributing to development of a dense support for extension of the epithelial skin layer [31]. CD90, a cell‐surface glycoprotein, mediates cell–cell and cell–matrix interactions, fibroblast activation, proliferation, and migration, thereby facilitating tissue remodeling and wound repair [19, 32]. Upregulation of *α*‐SMA reflects increased myofibroblast contractility and ECM production [20, 33]. The transcription factors ZEB1 and Snail promote fibroblast activation and mesenchymal transition [21–23], whereas *β*‐Catenin, a key WNT pathway component, contributes to fibroblast proliferation and ECM synthesis [34].

The stimulatory effect of nanoperlite appears closely linked to its distinct physicochemical characteristics, including nanoscale dimensions, large surface area, and SiO_2_‐rich composition [15]. These properties enhance cell–material interactions by increasing bioavailable surface contact, promoting adhesion, and facilitating intracellular uptake. Previous studies have shown that nanoperlite‐modified substrates improve surface hydrophilicity and cell adhesion, likely through elevated surface energy and protein adsorption [15]. Furthermore, the high SiO_2_ content plays a pivotal role in imparting bioactivity, similar to other silica‐based materials known to enhance fibroblast proliferation and ECM deposition. Collectively, these attributes create a favorable microenvironment that may prime TED OFs toward a more activated, fibrogenic phenotype.

Given that over 70% of nanoperlite′s composition consists of SiO_2_ [35], its biological activity is largely attributed to this component. SiO_2_ may exist alone or in combination with other metals, and silicon (Si) is recognized as an essential trace element, ranking among the most abundant in the human body after iron and zinc. SiO_2_‐based materials, including bioglass, mesoporous structures, and SiO_2_ nanoparticles, are widely used in drug delivery, tissue regeneration, and diagnostic applications. Accordingly, nanoperlite—due to its high SiO_2_ content—can be categorized within this class of bioactive materials [36].

There are different studies on the effect of SiO_2_‐based materials on fibroblasts. It has been shown that Si gel can regulate the expression level of bFGF in fibroblasts and improve the wound healing process by correcting the lack or abundance of growth factors and prevent the production of scar tissue [16].

It has also been demonstrated that bioglass can significantly increase the secretion of growth factors as well as the production of collagen I and fibronectin in fibroblasts and stimulate cell migration. Also, bioglass‐activated skin tissue engineering grafts can stimulate angiogenesis, increase the deposition of collagen and accelerate the contraction of the wound at the wound site and thus accelerate the healing process of the wound [37].

Beyond surface interaction, the biological effects of nanoperlite may be mediated through activation of canonical fibrotic signaling pathways. The marked upregulation of TGF‐*β* and *α*‐SMA suggests engagement of the TGF‐*β*/SMAD pathway, which governs fibroblast‐to‐myofibroblast transition and ECM synthesis [38]. Concurrently, the increase in *β*‐Catenin points to WNT/*β*‐Catenin pathway involvement, a key regulator of fibroblast proliferation, migration, and fibrogenesis [39, 40]. The upregulation of Snail and ZEB1—transcription factors linked to epithelial‐mesenchymal transition (EMT)—further supports activation of a profibrotic, mesenchymal phenotype [41]. Our bioinformatics network analysis further emphasized on these connections, demonstrating that the genes significantly modulated by nanoperlite (TGF‐*β*, CD90, *α*‐SMA, ZEB1, *β*‐Catenin, and Snail) form a highly interconnected network enriched for fibrogenesis‐related processes. Specifically, key first‐degree interactors identified within this network include *β*‐Catenin, and indicating direct involvement with the WNT/*β*‐Catenin pathway and TGF‐*β*‐SMAD. Other notable interactors, such as proteins involved in PI3K/AKT signaling cascade, also highlight broad engagement of pathways crucial for ECM deposition and fibrogenesis [42, 43]. Additionally, previous studies have shown that SiO_2_‐based nanoparticles may induce oxidative stress and activate MAPK and PI3K/AKT pathways, which converge with TGF‐*β* and WNT signaling and could amplify fibrogenic gene expression [44, 45]. Although we did not directly assess ROS or kinase activity in this study, these pathways present plausible mechanisms consistent with our findings and warrant future investigation.

According to the represented data, 1 *μ*g/mL nanoperlite could promote the expression of gene involved in ECM production, fibroblast activation, and migration. The observed upregulation of these markers suggests that nanoperlite treatment pushes TED OFs towards a more activated, fibrogenic, and myofibroblastic phenotype. This aligns with the desired outcome for generating robust fibrotic substrates for tissue engineering applications, indicating that nanoperlite potentially influences the core processes of fibroblast function and tissue remodeling.

Interestingly, as the concentration of nanoperlite was increased to 10 *μ*g/mL, the upregulation trend slowed, with only *β*‐Catenin showing significant increase. This observation suggests a potential threshold effect, wherein higher concentrations of nanoperlite may not proportionally enhance fibrogenesis, or may even trigger regulatory mechanisms that modulate fibrogenesis associated gene expression differently. These findings underscore the importance of considering dosage effects when exploring the therapeutic potential of nanomaterials in modulating cellular processes such as fibrogenesis.

### 4.1. Limitations and Clinical Translations

While our findings suggest that nanoperlite can enhance fibrogenic gene expression in thyroid OFs, translating these results into clinical applications requires careful consideration.

A limitation of this study is the use of OFs derived from a single TED patient. While this allowed for initial exploration of nanoperlite′s effects on an activated fibroblast phenotype, it may be associated with variability and limits the generalizability of our findings. Primary cell cultures from individual patients can exhibit diverse genetic characteristics and responses to treatment. Future studies must incorporate OFs from more TED patients to account for interpatient variability. Furthermore, considering the gender prevalence of TED (more common in women), including samples from female patients would be crucial. We also acknowledge the absence of a healthy control group, which would have been invaluable for understanding if nanoperlite specifically enhances the already activated phenotype of TED OFs or if it generally promotes fibrogenesis in normal fibroblasts. This comparison is essential for tailoring strategies for reconstructive surgery versus managing TED pathology.

Although silica‐based materials have shown promise in various biomedical contexts, the specific biocompatibility, immunogenicity, and degradation profile of nanoperlite—particularly in ocular tissues—remain to be fully characterized. The eye is an immune‐privileged site, but local inflammation or chronic immune responses to implanted materials could have severe consequences, including vision impairment or graft rejection. The small size and unique surface chemistry of nanoparticles can trigger specific immune pathways that differ from bulk materials. Therefore, comprehensive in vivo studies must assess inflammatory cytokine profiles, immune cell infiltration (e.g., macrophages and lymphocytes), and the long‐term foreign body response specifically within orbital and periorbital tissues following nanoperlite exposure.

Moreover, the retention, clearance, and potential accumulation of nanoperlite in periocular tissues present important safety considerations. Hence, before nanoperlite can be advanced for clinical use in ocular tissue engineering (e.g., for periorbital scaffolds or wound healing applications), comprehensive preclinical studies are essential. These should include cytotoxicity assessments in ocular cell types, animal models for biocompatibility and functional integration, and long‐term follow‐up for adverse tissue responses. We acknowledge these limitations and present our current findings as an early‐stage exploration of nanoperlite′s profibrotic potential. Further in vivo validation and safety profiling are necessary steps to support its translation to clinical applications.

Moreover, while our study focused on gene expression, it is crucial to investigate changes at the protein level and assess functional endpoints. Future work will include quantifying ECM components such as collagen, hyaluronic acid, and fibronectin deposition, as well as protein expression of key signaling molecules (e.g., phosphorylated SMAD2/3, nuclear *β*‐Catenin, and *α*‐SMA protein levels) using techniques like western blotting or immunofluorescence. This will provide direct evidence for pathway activation and confirm that the observed mRNA changes translate into functional protein outcomes, thereby strengthening the mechanistic claims.

## 5. Conclusion

In conclusion, our data demonstrate that nanoperlite at a concentration of 1 *μ*g/mL significantly upregulates the expression of genes involved in fibroblast activation, migration, and ECM production in TED OFs. This finding suggests that nanoperlite has the potential to be used for periorbital tissue engineering and eyelid reconstruction. However, further investigation into its dose‐dependent effects, long‐term safety, and clinical utility is warranted to optimize its application across regenerative strategies.

## Author Contributions

F.S‐J. and B.K. were involved in the project design. F.S‐J. and R.S. obtained the data and performed the experiments. B.K. provided the orbital samples for this study. All authors were involved in writing the draft article and revising the final version.

## Funding

This study was supported by Shiraz University of Medical Sciences (Grant# 30300). [Correction added on 17th July 2026, after first online publication: An incorrect grant number was initially included in the Funding section. This has now been updated and the correct grant number is displayed.]

## Ethics Statement

The protocol used in this study was approved by the Ethics Committee of Shiraz University of Medical Sciences (IR.SUMS.REC.1403.196) and written informed consent was obtained as part of the study′s support from Shiraz University of Medical Science.

## Consent

The authors have nothing to report.

## Conflicts of Interest

The authors declare no conflicts of interest.

## Supporting information


**Supporting Information** Additional supporting information can be found online in the Supporting Information section.. Table S1: Raw replicate data and statistical analysis for fibrogenesis‐associated gene expression (TGF‐*β*, CD90, *α*‐SMA, *β*‐Catenin, Snail, and ZEB1) in TED orbital fibroblasts following treatment with nanoperlite (1 and 10 *μ*g/mL). The table includes individual biological replicates, calculated mean and standard error of mean (SEM), and post hoc least significant difference (LSD) test *p* values compared with untreated control cells (set as 1), along with one‐way ANOVA statistics (*F*(2,6)).

## Data Availability

The data used to support the findings of this study are available from the corresponding author upon request.
